# Assessing the Quality of Community Jury Deliberations in Online and In‐Person Community Juries Using a Deductive Coding Framework

**DOI:** 10.1111/hex.70486

**Published:** 2025-11-13

**Authors:** Hanan Abukmail, Rebecca A. Dennison, Juliet A. Usher‐Smith, Lily C. Taylor

**Affiliations:** ^1^ Primary Care Unit, Department of Public Health and Primary Care University of Cambridge Cambridge UK; ^2^ London School of Hygiene and Tropical Medicine, Faculty of Epidemiology and Population Health London UK

**Keywords:** community jury, deliberation, deliberative quality, online research, qualitative research

## Abstract

**Introduction:**

Community juries require participants to engage in group deliberations, seeking to reach a consensus or verdict on the research question(s). Community juries have traditionally taken place in‐person, although holding them online or in a hybrid format is increasingly common, particularly following the COVID‐19 pandemic. In order for the community jury method to be used successfully, it is crucial to ensure that the goals of the deliberative process are accomplished.

**Objective:**

To assess the quality of unfacilitated deliberation in community juries held either online or in‐person.

**Methods:**

We conducted a secondary analysis of three community juries exploring the public acceptability of novel cancer screening and referral policies informed by personal risk. Two juries were held online, and one was held in‐person. We analysed the deliberations via framework analysis, using a previously developed deductive coding framework that addresses the theoretical goals underlying the deliberative process. Participants also completed post‐jury surveys including questions relating to their experiences of the online or in‐person setting.

**Results:**

Nine participants attended the in‐person jury, and a total of 15 attended online. Both online and in‐person juries achieved all six deliberative goals to some extent with juries in both settings able to participate in successful deliberation and produce well‐informed recommendations from a societal perspective.

**Conclusion:**

These findings suggest that community juries delivered both online and in‐person have the potential to facilitate high‐quality deliberations, and both settings can be used to successfully conduct community juries.

**Patient or Public Contribution:**

Four PPI representatives, who represented people with different characteristics, including a personal history of cancer, were involved throughout this research. Their contribution included the formation of the research question for the funding application, design of participant‐facing aspects of the research, such as presentations used within the juries, and creation of lay summaries of findings.

## 1 Introduction

Community juries are a relatively novel method for gaining public insight into topics that require consideration of both community values and scientific evidence [1, 2]. The aim is to elicit participant perspectives as they attempt to reach a verdict or consensus on a given research question, after being educated about the topic [1]. Community juries are a deliberative democratic method, meaning that participants should engage in equal and respectful communication involving their preferences, values and interests [3]. The method encourages participants to expand their thinking beyond their individual interests to consider the perspective of society as a whole and the needs of the population, to generate informed and objective recommendations [1, 4]. They are of particular value in determining an informed public opinion on topics that require consideration of scientific concepts, ethical principles and the balancing of harms and benefits [4, 5]. Public involvement through community juries has been shown to benefit the quality of recommendations and decision‐making for public policymaking, and to strengthen trust in the resulting policy decisions [2, 4].

The COVID‐19 pandemic necessitated a shift towards online research methods as in‐person, face‐to‐face data collection was no longer permitted due to social distancing measures [6, 7]. Online data collection has continued beyond the pandemic, and this approach has a multitude of benefits for participants and researchers alike. For example, online research may be more convenient by allowing individuals to participate from a location of their choice, enabling them to feel more comfortable discussing personal stories and perspectives and allowing for a geographically diverse sample [8]. Travel costs and time are also minimised, which is particularly relevant for participants who work, have caregiving responsibilities or disabilities [9]. Many of these benefits extend to researchers. Conversely, online research restricts participation to those with suitable devices and internet access, and may reduce accessibility for those with low digital literacy or specific disabilities [10]. The opportunity for informal interaction during breaks is also lost with online methods.

It is important to understand how the two approaches fare in terms of the depth and quality of the data generated. Research on this topic is sparse, but previous studies investigating other qualitative methods have reported that data obtained online are comparable to those obtained in‐person [11, 12, 13, 14, 15]. For example, studies of focus groups and individual interviews reported no difference in the mean number of qualitative codes or disclosure of sensitive information between online and in‐person research. Interview participants reported similar levels of comfort between online and in‐person settings; however, participants in the online focus groups were significantly less satisfied with their experience [11]. A 2024 comparison of online (discussion forum) and in‐person focus groups for people affected by multiple sclerosis also reported a high degree of thematic overlap between the two modalities. In‐person discussions tended to include more detailed input but were also more likely to go off‐topic or be dominated by one person [13].

Methodological choices have the potential to impact the quality of discussion, which could be argued to have a greater significance for the success of deliberative methods like community juries in which participants aim to reach consensus than for focus groups [2]. Scott et al. identified the underlying goals of community jury deliberations through literature searching and generated a deductive coding framework to assess the quality of such deliberations. These goals focus on the unfacilitated deliberation within community juries. The goals are the following: expressing preferences and values, reciprocal interactions and consideration of alternative views, use of expert information to enhance participant knowledge, producing thoughtful and evidence‐based recommendations and considering a societal perspective [5]. The overall quality of deliberation can be measured by how effectively each of the goals is met. Assessing whether community juries held in different settings accomplish these goals is crucial to informing how future juries are designed and implemented. We therefore aimed to assess the quality of unfacilitated deliberation between online and in‐person community juries using this deductive coding framework [5]. Secondarily, we explored participant preferences and included considerations for researchers, policymakers and public engagement practitioners conducting juries in the future.

## 2 Methods

### 2.1 Data Source

This is a secondary analysis of data collected in 2023 from three community juries that aimed to understand public receptiveness to novel risk‐based approaches for cancer prevention and early diagnosis. Ethical approval was obtained from the University of Cambridge Psychology Research Ethics Committee (PRE.2023.002). Detailed methods and findings are reported separately [16].

### 2.2 Community Jury Structure

Two of the community juries were held online using Zoom videoconferencing software, and one jury was held in‐person in Cambridge, England, in a university conference room in which participants were seated around a table with access to audiovisual equipment. The duration of the juries was up to eight hours, with the online juries held across two consecutive days and the in‐person jury in a single full‐day session. All interactive sessions were recorded using Zoom after obtaining written consent from participants, and the recordings were then transcribed verbatim.

Briefly, the first session began with pre‐recorded expert presentations followed by question and answer (Q&A) sessions, during which the experts joined all three juries via Zoom. Two researchers with previous experience of community juries and qualitative research then facilitated a discussion to enable jurors to think through the topic. Finally, the jurors took part in an unfacilitated deliberation whereby the research questions, Table S1, were explained verbally as well as provided in writing using the Zoom chat function for online participants and on a screen for those in person. Participants were asked to nominate a representative to coordinate the deliberation, take notes and deliver the final verdict. During the unfacilitated deliberation, the researchers either left the room or turned their cameras and microphones off.

Participants completed pre‐ and post‐jury questionnaires to collect sociodemographic data and provide feedback on their experience of the community jury and the respective setting.

### 2.3 Participants and Recruitment

Each community jury comprised 7–9 members of the public recruited via iPoint Research Ltd, a market recruitment company. Participants were purposively sampled based on age, sex, ethnicity, socioeconomic status and geographical location. Participants were aged 21–79 years and were fluent in English. Online participants were required to have a suitable device with internet connectivity, a microphone and camera, and to attend a technology check with the recruiter before taking part. In‐person participants needed to be able to travel to the site of the jury. Individuals were allocated to the juries based on their availability and to ensure a range of demographic characteristics.

### 2.4 Analysis

The written transcripts from unfacilitated deliberations and feedback sessions were analysed by researchers with previous experience in qualitative analysis (H.A.) and (L.C.T.) using qualitative framework analysis in NVivo 15 software (Lumivero) [17]. We carried out deductive coding using a previously developed framework for assessing the quality of jury deliberations that includes six goals relating to the deliberative aspect and the final recommendation (Table 1). All transcripts were double‐coded [5]. The researchers qualitatively determined whether each goal was achieved through iteratively reviewing the transcripts. Goal achievement was categorised as very well achieved, somewhat achieved or not achieved based on an evaluation of whether/how well each component of the goal was fulfilled. Subthemes were also generated inductively in cases where multiple concepts were identified within a single goal. A matrix was used to summarise the themes and enable comparison across goals and between jury settings (Table S2).

**Table 1:** Deductive coding framework for assessing the quality of community jury deliberations developed by Scott et al. [5].

Goal	Associated questions	Explanation
G1. Express values and preferences of participants	Does the CJ deliberation refer to individuals' values and preferences?	The jurors raise values and preferences (e.g., autonomy, transparency and greater good) during the deliberation
G2. Reciprocal interactions and consideration of alternative views	Did the jurors engage with each other's perspectives during the deliberation?	The jurors engage with each other's points, views and arguments (e.g., via clarification, agreement and building on each other's points)
G3. Enhance participants' knowledge	Does the CJ deliberation reference information from the experts?	The jurors cite points that were made by experts during expert presentations
G4. Produce thoughtful, well‐informed solutions	Does the CJ recommendation directly address the charge that the CJ was given?	The jurors reach a recommendation that directly responds to the research question that the jury aimed to address
	Does the information provided by the experts enrich the deliberation?	The jurors engage with the points made by the experts during the presentations (e.g., a juror raises a point, and others engage with it by challenging, affirming, negating or clarifying)
	Has the CJ reached a clear and identifiable recommendation?	The jurors reach a recommendation that can be clearly identified
G5. Provide reasons for recommendation	Do the jurors provide justification(s) for the recommendation they reached?	Reasons are offered in support of the recommendation reached
G6. Produce recommendations from a societal (rather than individual) perspective	Does the CJ deliberation reflect a societal (rather than individual perspective)?	The jurors differentiate between the decision they would make for themselves personally and the decision they would make for the community as a whole

Abbreviations: CJ, community jury; G, goal.

## 3 Results

### 3.1 Participant Characteristics

A total of 24 participants attended the community juries; nine participants attended in‐person, eight and seven attended the first and second online juries, respectively (Table 2). The ethnicity of most participants was White (*n* = 18, 75%). Each jury was attended by both females and males with a range of age groups and educational levels.

**Table 2:** Participant characteristics.

	Online (%)	In‐person (%)	Total (%)
*Total N*	15	9	24 (100.0)
*Age (years)*			
21–39	5 (33.3)	3 (33.3)	8 (33.3)
40–49	2 (13.3)	3 (33.3)	5 (20.8)
50–59	3 (20.0)	1 (11.1)	4 (16.7)
60–79	5 (33.3)	2 (22.2)	7 (29.2)
*Sex* a			
Female	6 (40.0)	4 (44.4)	10 (41.7)
Male	9 (60.0)	5 (55.6)	14 (58.3)
*Simplified ethnicity*			
Asian	1 (6.7)	1 (11.1)	2 (8.3)
Black	2 (13.3)	0 (0.0)	2 (8.3)
Mixed or multiple ethnic group	2 (13.3)	0 (0.0)	2 (8.3)
White	10 (66.7)	8 (88.9)	18 (75.0)
*Social grade*			
Middle class	7 (46.7)	5 (55.6)	12 (50.0)
Working class	8 (53.3)	4 (44.4)	12 (50.0)
*Education level*			
Not completed A levels, further education or equivalent	4 (26.7)	3 (33.3)	7 (29.2)
Completed A levels, further education or equivalent	5 (33.3)	1 (11.1)	6 (25.0)
Completed a bachelor's or postgraduate degree	6 (40.0)	5 (55.6)	11 (45.8)

^a^ For all participants, gender identity corresponded to sex as registered at birth.

### 3.2 Analysis of Community Jury Deliberations Using the Deductive Coding Framework

The extent to which each jury setting achieved the deliberative goals is summarised in Table 3.

**Table 3:** Qualitative assessment of the extent to which juries in each setting achieved the goals.

Goal	Online assessment	In‐person assessment
G1. Express values and preferences of participants	Very well achieved ✓✓	Somewhat well achieved ✓
G2. Reciprocal interactions and consideration of alternative views	Very well achieved ✓✓	Somewhat well achieved ✓
G3. Enhance participants' knowledge	Somewhat well achieved ✓	Somewhat well achieved ✓
G4. Produce thoughtful, well‐informed solutions	Very well achieved ✓✓	Somewhat well achieved ✓
G5. Provide reasons for recommendation	Very well achieved ✓✓	Somewhat achieved ✓
G6. Produce recommendations from a societal (rather than individual) perspective	Very well achieved ✓✓	Somewhat achieved ✓

#### 3.2.1 Goal 1: Express Values and Preferences of Participants

Participants across both juries expressed their values and preferences throughout the deliberation. The online jurors achieved this goal comprehensively by consistently sharing more details about their opinions, including nuanced values and caveats, whereas the in‐person jurors referred to their values and preferences less frequently and in less detail. Instead, a show of hands was sometimes used to indicate overall preference.

I'm also a yes, I think it's acceptable to use the data. I feel like there should maybe be a limitation on the sources or a clear definition of the sources that will be used, which is pretty much what everyone's said to an extent.

—P22, online

Yes, so genetics and polygenetic risk, so that's newly collected and individual data. So, maybe we do a show of hands, who's in favour of that being used.

—P18, in‐person

The online jurors also discussed a wider range of values, including justice, freedom, security and privacy, and multiple participants acknowledged that other peoples' opinions may differ from their own, helping to create a sense of safety when expressing contradictory values or preferences.

#### 3.2.2 Goal 2: Reciprocal Interactions and Consideration of Alternative Views

In general, participants in both juries were able to engage with each other's perspectives and asked for clarification where necessary. The online participants demonstrated particularly strong reciprocal interactions and specifically invited quieter jury members to share their views.

Are you happy with that P4? You're keeping very quiet.

—P6, online

Online jurors also showed comfort in vocalising alternative or contradictory views and demonstrated their understanding of the research question by explaining things to other members of the group. On the other hand, in‐person participants struggled to achieve reciprocity due to a single dominant voice often disproportionately shaping the narrative. This may have resulted in participants feeling inhibited to explore personal opinions and ethical concepts in greater depth due to the frequency of interruption and the leading views of one individual. There was greater evidence of both over‐speaking and interrupting in the in‐person setting, largely, but not entirely perpetrated by the dominant individual.

So, this guy who comes in with lower back pain, I would think that they would look at the age, they would look at the weight, look at the job – [interrupted].

—P17

But my point is, a minute ago, I'm not going to – [interrupted].

—P18

Yeah, that's fine.

—P17, in‐person (dialogue between P17 and P18)

Participants in both the online juries assumed the role of ‘devil's advocate’ to look beyond their individual perspective and also explored alternative opinions and scenarios that did not apply to them personally, but that they recognised people in wider society might have.

But then, is it a slippery slope? Sorry, I'm playing devil's advocate here…

—P6, online

Online participants also validated each other's perspectives by verbally agreeing or confirming. This helped to promote a strong group dynamic in which participants were able to share alternative views and feel comfortable in their interactions. This mechanism of validation was also present in the in‐person jury, albeit to a lesser extent. However, it is possible that non‐verbal cues like nodding were utilised in the same way but were not captured by the verbal transcripts.

All juries were asked to nominate a representative responsible for feeding back the verdict at the end of the deliberation. Across the three juries, the representative also assumed a facilitatory role and often attempted to moderate the discussion to keep the group on track and ensure a consensus recommendation was reached. The online representatives were more successful in steering the discussion and encouraging inclusive deliberation. The in‐person representative made multiple attempts at facilitating but was less successful, largely due to repeated interruption and stronger voices taking control.

#### 3.2.3 Goal 3: Enhance Participants Knowledge

Participants across all three juries referenced information delivered by the experts to support the deliberation. Online participants referred to the exemplars discussed at the start of the juries and used these when considering the acceptability of different types of data. Several participants considered the ethical principles introduced by an expert presenter, using these to debate whether the innovations could be considered ethically sound.

It was also agreed that as a democratic society that would be the right thing to do, and, you know, in regards to justice one of the four areas that were discussed as well, that right to choose was very important.

—P22, online

Examples of statistics included in the expert presentations were also used to enhance the online deliberations and ensure the discussion remained grounded in evidence.

I think going back as well, a 2% high risk compared to 98% low risk, it's a bit of a non‐brainer, isn't it, cost‐wise?

—P2, online

Online participants referred back to the exemplars on multiple occasions and helped to explain and remind one another what these entailed when necessary.

The examples given were lung cancer for pollution and skin cancer based on climate but I guess it could include other things. Couldn't it?

—P6

There was something to do with your home. Not your postcode but your home as well. It was something about people should be getting electric ovens rather than using gas. It was in relation to health as well.

—P10, online (dialogue between P6 and P10)

In‐person participants also referred to the exemplars provided by the experts, including wearable devices and the use of novel diagnostic tests.

They've done the testing. They've done all the [inaudible]. It's been signed off by medical professionals and all the other stuff. It's been trialled for five years. And now they're going to start rolling out the [Cyto]sponge.

—P18, in‐person

They demonstrated understanding of the process of risk assessment and risk stratification through their dialogue and used this to rationalise their argument.

But if you have a symptom and you're technically low risk for everything else, you're still going to be on their radar. It's not going to be the same with – like so when it said, you're 40, you have no underlying family issues, and you're a healthy weight, but there might be a slight… then you move up that scale. That's all that's saying. It's still risk assessing, but they might use more data to narrow that field down even more.

—P18, in‐person

Participants across all juries also drew on information derived from personal anecdotes, experiences with screening or healthcare and evidence from news media to enrich the deliberations beyond the data provided by the experts. However, online participants shared this type of information more frequently, appearing more comfortable disclosing personal details than participants in the in‐person jury.

But on the other hand, my husband has lots of follow‐ups via phone. He doesn't actually have to go to a hospital. He has the follow‐up by phone. So, that saves a lot of time and effort as well.

—P2, online

#### 3.2.4 Goal 4: Produce Thoughtful, Well‐Informed Solutions

Online and in‐person participants considered the research questions as set out in the jury charge. In the online setting, the jurors successfully addressed the jury charge by working systematically through the research questions and covering each one in turn.

Do we think it's acceptable, in general, to use data? We were happy with the in general use of data.

—P1, online

All the questions contained within the jury charge were adequately addressed during the allotted time, and the jurors were able to reach clear and identifiable recommendations based on the information provided at the jury's outset and their own opinions.

The in‐person jurors also deliberated on the questions set out in the jury charge. However, the deliberation was noticeably skewed towards some questions more than others. For example, they spent a long time debating the correct interpretation of the first research question, rather than answering it, and failed to leave enough time to discuss the remaining questions in the same level of detail.

But the question I'm reading is, they're saying ‘Are you more or less comfortable with this?’ Are they still using the risk basis, like we've been talking, to assess, so if you've got symptoms whether we now take you to the next stage. That's what worries me… I'm still uncomfortable with the way they're asking the question, but I accept that is it… [interrupted].

—P14

Well, that's what I read it as.

—P18, in‐person (dialogue between P14 and P18)

As a result of this debate, the jury failed to provide a clear recommendation for one of the questions laid out in the jury charge. This was evident in the final feedback session, during which participants continued to exchange views rather than delivering a consensus recommendation. Nevertheless, they continued working through the remaining questions in a systematic way and established thoughtful and informed recommendations for these.

#### 3.2.5 Goal 5: Provide Reasons for Recommendations

Online participants provided reasoning during the deliberative process through the reciprocal sharing of views and preferences as well as delivering their final recommendations alongside the rationale. Jurors were able to go into detail about the process of decision‐making they had gone through as part of the deliberation when probed by the expert in the final feedback session.

We're okay with that, postcode information but we felt there were socioeconomic considerations that would need to be taken into account. One example we had was if you live in Manchester, London or Birmingham, you're in smoky town centres or whatever, you're probably more likely to get cancer.

—P1, online

The in‐person participants appeared to struggle to fully provide the reasoning underpinning their values and preferences due to the frequent interruptions during the deliberation. They were able to justify their final recommendations to some extent but neglected to provide as much detail as the online participants.

#### 3.2.6 Goal 6: Produce Recommendations From a Societal (Rather Than Individual) Perspective

Both groups of jurors achieved this goal well, although the online participants devoted more of their deliberations to societal considerations. Both online and in‐person juries considered the impact of their recommendations on the wider health service in terms of funding and capacity. The deliberations also explored the potential impact of the example interventions on different subgroups of the population. For example, the online participants discussed people who are symptomatic compared with those who are asymptomatic, older individuals as well as younger ones, socioeconomic differences and the potential widening of inequalities.

We mentioned about ethics in terms of I guess the AI and maybe there being a lens that… you know, an unconscious bias where the questioning is… the commands in AI have been programmed in a particular way that maybe doesn't represent the overall population so the outcomes that are being derived from the AI maybe exclude or maybe don't take into account certain things.

—P23, online

They discussed factors such as liberty and freedom of choice and considered the importance of preserving these as part of maintaining a democratic society. The in‐person deliberation explored many of the same concepts but in less detail, possibly as a result of having less time to spend deliberating after debating the interpretation of the first research question.

### 3.3 Participant Reflections

Participant feedback is summarised in Table 4. Online and in‐person participants both rated the overall experience positively and felt that the respective setting helped them to participate, understand the information and engage with other participants. No statistical differences were observed between settings. Participants from the in‐person jury reflected that the in‐person setting facilitated concentration and interpersonal interaction:

It felt more real, part of a team of people representing the community; I feel people engaged well and worked well together despite differences of opinion and disagreements.

—P13, in‐person

**Table 4:** Participants' experiences of participating in the community juries according to setting.

	Online	In‐person	*P* value
*Total N*	15	9	
*Experience of the CJ* a			
Overall experience	10 (9–10)	9 (8–10)	0.144
Anticipated experience in the opposite setting	9 (8–10)	6 (5–7)	0.025
*How well the setting helped them to…* b			
Participate in and contribute to the jury	5 (4–5)	4 (4–5)	0.283
Learn and understand the information	5 (4–5)	4 (4–5)	0.211
Feel a sense of community with the other participants	4 (3.5–5)	4(4–5)	0.721
*Preferred setting for future CJs* c			
No preference	4 (26.7)	0 (0.0)	NA
Online	5 (33.3)	0 (0.0)	
Hybrid	4 (26.7)	2 (22.2)	
In‐person	2 (13.3)	7 (77.8)	

Abbreviation: CJ, community jury.

^a^ Rated 0 = worst to 10 = best (median [interquartile range]; Kruskal–Wallis test for difference).

^b^ Rated 1 = not at all well, 2 = slightly well, 3 = moderately well, 4 = very well and 5 = extremely well (median [interquartile range]; Kruskal–Wallis test for difference).

^c^ *N* (%).

Similarly, participants from the online juries identified benefits to taking part online, including the convenience and comfort derived from participating from their own space and a sense of safety when sharing personal perspectives:

It's easier to talk online, you feel less self‐conscious than you do face‐to‐face, so more likely to voice your opinion.

—P6, online

Participants who completed the jury online were more positive about the prospect of an in‐person jury than participants who completed the jury in‐person were about attending an online jury. When asked about their preferred setting for future juries, the majority of those who participated in‐person reported that they would prefer to participate in‐person again (*n* = 7, 77.8%). Online participants expressed more varied preferences, with four individuals stating no preference (26.7%) and nine participants wanting at least parts of a future jury to be held online (60.0%).

## 4 Discussion

Following an increase in the use of videoconferencing in research over recent years, it is essential to ensure the quality of community jury deliberations is maintained in the virtual setting. In this assessment of juries conducted with the same format and research question online and in‐person, participants' self‐reported experiences and their preferences towards the setting were largely comparable between the two groups. All participants rated their experience of the jury highly positively, and there were no statistical differences between how well the jury setting helped participants to learn, participate or cultivate a sense of community with their fellow jurors. The quality of deliberation was high in both the online and in‐person juries, although the online deliberation in these examples was more successful overall. As a result, we suggest that researchers can feel confident choosing either setting in future research and have ended this paper with considerations for conducting deliberations that can be applied to both online and in‐person juries to support the goals of community juries, as well as reflections on the deliberation quality assessment framework.

Prior research has compared other qualitative research methods such as interviews and focus groups in this way. Our finding that both online and in‐person deliberations were successful overall and achieved all the goals to some extent is similar to previous studies comparing qualitative research conducted in a virtual setting with traditional in‐person methods. In those studies, both approaches have been found to be effective and produce similar data [11, 12, 13, 14, 17], and a comparison of interviews and focus groups between online and in‐person research reported that the overall data generated were similar regardless of the research setting [11].

Nevertheless, one of those previous studies also reported that the social dynamic varied between settings [11]. It has also previously been observed that in‐person qualitative research is more likely to be disrupted by a dominant individual, causing challenges for the moderator, as was observed in the in‐person community jury [13, 14]. Similarly, in‐person group discussion has been found to generate more irrelevant or off‐topic discourse [13, 14]. Based on our observations of our community juries, it may be the case that the impact of the virtual environment, such as the one‐speaker‐at‐a‐time dynamic and visual cues that highlight the current speaker, help ensure that all participants have an equal opportunity to contribute, potentially mitigating the dominance of louder voices.

In terms of participant feedback, one study found that online participants were significantly less satisfied with their experience of online focus groups compared with those who attended in‐person, despite no differences between perceptions of rapport, safety, comfort or convenience. The authors speculate that technological difficulties contributed to this difference, including issues with internet connectivity, as well as difficulty warming to other participants online [11]. Online jurors reported very positive experiences of taking part in our study, as well as comparable levels of participation, learning/understanding and a sense of community.

This is one of the first studies to apply the quality assessment framework developed by Scott et al., in practice [5]. We found that the framework provides useful domains for conceptualising what constitutes quality in the context of deliberative research and encourages researchers to consider quality in terms of both the deliberation itself and the resulting recommendations. However, the framework goals overlap to some degree, which made it challenging to apply in practice; it was particularly noticeable how Goal 2 (Reciprocal interactions and consideration of alternative views) closely aligned with all the others. We also found overlap between Goals 3 and 4, which both encourage interaction with the knowledge provided by the experts. Researchers using the framework in future could better operationalise goals relating to the use of external information by limiting the use of expert‐derived information to a single goal and potentially combining this with the use of pre‐existing personal knowledge, such as from the news or individual experiences. Finally, the framework lacks guidance on how to measure the achievement of each goal individually and how or whether to aggregate the goals into an overall measure of quality. In this study, we did not generate a single measure of quality; instead, we explored how well each goal was met individually and synthesised overall findings about the respective quality of deliberations in each setting via the discussion.

### 4.1 Strengths and Limitations

A key strength of this study is the use of an established peer‐reviewed deductive framework specifically designed for the evaluation of community jury deliberations. Additionally, all three juries were conducted with the same study team and on the same topic. All factors except for the setting were kept as constant as possible to enable comparison, and this analysis was led by researchers who were not involved, or only peripherally involved, in the main study to promote unbiased interpretation. Nevertheless, the researchers leading the analysis were aware, which setting each deliberation took place in. A final strength is the timeliness of this research. Online research has expanded considerably since 2020, and this study is the first to begin to address the knowledge gap around the impact this may have on the quality of deliberative measures.

A limitation of this study is the small number of juries that were analysed, particularly for the in‐person setting with only a single jury compared with two online juries. The juries were facilitated by a single research team who may have been better at facilitating in one particular setting. An additional potential limitation is the skill and comfort of the jury representatives, who assumed a quasi‐facilitator role during the unfacilitated deliberation. Additionally, the in‐person jury was held over 1 day rather than being split into two consecutive days like the online juries. This means that in‐person participants may have felt fatigued towards the end of the process, which included the unfacilitated deliberation and feedback session. Importantly, it is also not possible to say whether the differences observed were due to the setting or due to differences in juror characteristics or personalities. For example, the dominant character in the in‐person jury may have been equally disruptive had they participated online. Furthermore, the nature of classifying how well a goal has been achieved is inherently subjective. Finally, this analysis was based solely on written transcripts, meaning that any non‐verbal interactions were absent and that the quality of the in‐person transcript was reduced compared with the online transcripts due to the challenge of recording multiple voices at once.

### 4.2 Considerations for Conducting Future Community Juries

Positively, our results suggest that researchers should feel confident conducting deliberative research online as the virtual setting does not appear to inhibit the deliberative process. We would encourage researchers to consider using both settings to maximise diversity and accessibility and benefit from the relative strengths of both approaches whilst preserving the integrity of the deliberation. To support the quality of deliberation in both settings, we suggest researchers consider ways to mitigate dominant voices, to support time management during unfacilitated deliberation and to ensure that the research questions are fully understood by all members of the jury.

Ways to mitigate a dominant voice in any setting include nominating an alternative member of the jury as spokesperson and establishing clear ground rules for communicating at the outset. These rules could include not interrupting or over‐speaking and giving all participants an opportunity to be heard. The in‐person participants in this study also demonstrated poor time management, losing sight of the overall objective of the deliberation and becoming bogged down in the semantics of a single question. To avoid this in future, we suggest allocating an approximate time to spend on each question or having the researchers check in on the jurors at set intervals to monitor the process. Similarly, to limit conflicting interpretations of the jury charge, researchers should explain the research question(s) in full and confirm understanding with the jurors before commencing unfacilitated deliberation. The goals of the deliberation should also be made clear at the outset. Analysing video recordings of the deliberations where possible may help to capture non‐verbal interactions that may otherwise be missed from using written transcripts alone.

### 4.3 Conclusion

We have shown that both online and in‐person settings can be used to conduct community juries with high‐quality deliberations that successfully address the jury charge and produce consensus recommendations from a societal perspective. Researchers conducting community juries in future should be mindful of mitigating dominant personalities and extraneous discussion in in‐person settings in particular.

## Author Contributions

**Hanan Abukmail:** conceptualisation, data curation, formal analysis, software, methodology, writing – original draft, writing – review and editing. **Rebecca A. Dennison:** conceptualisation, data curation, funding acquisition, methodology, supervision, writing – review and editing. **Juliet A. Usher‐Smith:** conceptualisation, funding acquisition, methodology, supervision, writing – review and editing. **Lily C. Taylor:** conceptualisation, data curation, formal analysis, software, methodology, supervision, writing – original draft, writing – review and editing.

## Ethics Statement

Ethical approval was obtained from the University of Cambridge Psychology Research Ethics Committee (PRE.2023.002).

## Conflicts of Interest

The authors declare no conflicts of interest.

## Supporting information


**Supplementary Table 1:** Questions for unfacilitated deliberation in the CJs. **Supplementary Table 2:** Framework matrix summarising the qualitative data across deliberative goals for online and in‐person juries.

## Data Availability

Pseudo‐anonymised transcripts and study materials (protocol, participant information sheet, consent form, participant information pack, facilitated discussion topic guide and questionnaires) are available via the University of Cambridge Data Repository: https://doi.org/10.17863/CAM.107283. Formal requests for access will be considered via a data‐sharing agreement that indicates the criteria for data access and conditions for research use and will incorporate privacy and confidentiality standards to ensure data security.
