# Supplementary material for: Assessing the Quality of Community Jury Deliberations in Online and In‐Person Community Juries Using a Deductive Coding Framework
Source: Health Expect. 2025 Nov 13;28(6):e70486. doi: 10.1111/hex.70486 (PMC12614172; doi:10.1111/hex.70486)
Supplement: Supplementary file 1 — Supplementary Table 1: Questions for unfacilitated deliberation in the CJs. Supplementary Table 2: Framework matrix summarising the qualitative data across deliberative goals for online and in‐person juries. [file HEX-28-e70486-s001.pdf]

Assessing the quality of community jury deliberations in online and in-person community juries using a deductive coding framework

Supplementary material

Supplementary table 1. Questions for unfacilitated deliberation in the CJs.

**Questions**
Q1. Do you think it is acceptable in general to use data from a range of sources to assess cancer risk and use that risk to determine access to healthcare?
Q2. Does it make a difference whether people have symptoms or not? i.e., are you more or less comfortable about using risk-based approaches such as those discussed today to assess people with cancer symptoms, compared with those without symptoms (screening)?
Q3. Does it make a difference what data are used? i.e., are you more comfortable with using some of the examples that we‘ve shown you today than others? Are you more or less comfortable about using data that is automatically collected (such as loyalty cards, medical records, or postcode), compared with data that requires action by individuals to collect (such as self-tracking apps)?
Q4. Do you think people need to have the option to opt out of the use of data being used in this way? If so, how would you handle those individuals?

CJ – community jury; Q – question

Supplementary table 2. Framework matrix summarising the qualitative data across deliberative goals for online and in-person juries.

	**G1 Express values and preferences of participants**	**G2 Reciprocal interactions and consideration of alternative views**	**G3 Enhance participant's knowledge**	**G4 Produce thoughtful, well-informed solutions**	**G5 Provide reasons for recommendation**	**G6 Produce recommendations from a societal perspective**	**Other**
**Online**	Participants consistently express their personal preferences and values. These include nuanced or caveated expressions of values. Participants discuss values such as fairness/justice, security, privacy, freedom of choice. Several participants also acknowledge their preferences as their own, understanding the others may feel differently.	Participants have strong reciprocal interactions and frequently considered alternative viewpoints/ opinions. They also assumed the role of 'devil's advocate' to explore alternative opinions that they did not personally hold, but that others in wider society might have. Participants specifically invited quiet jury members to share their views. Jurors seem comfortable voicing alternative opinions and asking questions/providing explanations to each other.	Participants refer to the exemplars given by the experts, including when considering which types of data to use. One participant refers directly to the expert presentation. Jurors draw on their own knowledge to enhance the deliberation, including examples of news articles and NHS health campaigns.	Participants consider detailed and diverse aspects of the jury charge. Jurors successfully address the jury charge by working systematically through the research questions. Jurors reach a clear and identifiable recommendation for all research questions.	Participants provide reasoning when sharing individual values and preferences and the final recommendations were delivered with a rationale.	Participants consider the impact on the health service financially and in terms of capacity. They consider the impact on specific population subgroups, such as people who are asymptomatic /symptomatic. Discussion about liberty and freedom of choice as part of a democratic society.	Jurors have good rapport, respectfully managing the logistics of the deliberation, sharing personal stories and anecdotes to enhance discussion, and laughing/joking together.

**In-person**	Participants express values and preferences to some degree and use show of hands to indicate acceptability.	Jurors engage with each other's perspectives and ask questions to clarify. Over-speaking and interrupting make it challenging for opinions to be truly heard. One individual dominates the discussion. A verdict was not reached for all research questions, meaning reciprocal interactions continued into the feedback portion of the jury.	Participants make some reference to the exemplars given and use the Cytosponge and Fitbit as an example. Some jurors had difficulty recalling all the innovations.	Participants consider the research questions as set out in the jury charge however deliberation was skewed and consensus was unable to be reached for one question. Jurors successfully addressed the charge for the other research questions and worked through the exemplars systematically.	The recommenda-tions were delivered with explanations. Participants struggle to provide reasoning for their own values and preferences.	Participants successfully considered the charge from a societal perspective, considering the impact on different population subgroups, such as older vs younger. They also considered financial implications for the health system.	There was a lot of confusion and debate between jurors as to the meaning of the research questions and what they were expected to do to address the jury charge. Some evidence of using personal stories to enhance the deliberation or provide examples.
